# Cellular chaperones and folding enzymes are vital contributors to membrane bound replication and movement complexes during plant RNA virus infection

**DOI:** 10.3389/fpls.2012.00275

**Published:** 2012-12-06

**Authors:** Jeanmarie Verchot

**Affiliations:** Department of Entomology and Plant Pathology, Oklahoma State UniversityStillwater, OK, USA

**Keywords:** RNA virus replicase, cellular chaperones, unfolded protein response, virus intercellular movement, HSP70 heat-shock proteins, HSP90 heat-shock proteins, DNAJ homologs

## Abstract

Cellular chaperones and folding enzymes play central roles in the formation of positive-strand and negative-strand RNA virus infection. This article examines the key cellular chaperones and discusses evidence that these factors are diverted from their cellular functions to play alternative roles in virus infection. For most chaperones discussed, their primary role in the cell is to ensure protein quality control. They are system components that drive substrate protein folding, complex assembly or disaggregation. Their activities often depend upon co-chaperones and ATP hydrolysis. During plant virus infection, Hsp70 and Hsp90 proteins play central roles in the formation of membrane-bound replication complexes for certain members of the tombusvirus, tobamovirus, potyvirus, dianthovirus, potexvirus, and carmovirus genus. There are several co-chaperones, including Yjd1, RME-8, and Hsp40 that associate with the bromovirus replication complex, pomovirus TGB2, and tospovirus Nsm movement proteins. There are also examples of plant viruses that rely on chaperone systems in the endoplasmic reticulum (ER) to support cell-to-cell movement. TMV relies on calreticulin to promote virus intercellular transport. Calreticulin also resides in the plasmodesmata and plays a role in calcium sequestration as well as glycoprotein folding. The pomovirus TGB2 interacts with RME-8 in the endosome. The potexvirus TGB3 protein stimulates expression of ER resident chaperones via the bZIP60 transcription factor. Up-regulating factors involved in protein folding may be essential to handling the load of viral proteins translated along the ER. In addition, TGB3 stimulates SKP1 which is a co-factor in proteasomal degradation of cellular proteins. Such chaperones and co-factors are potential targets for antiviral defense.

## INTRODUCTION

Positive-strand RNA viruses are among the largest group of viruses infecting plants worldwide and contribute to some of the most critical issues in agriculture. Two types of cellular alterations that are essential for (+) strand RNA virus replication and cell-to-cell movement include: (1) discrete and well characterized changes in the endomembrane architecture, and (2) the recruitment of host factors into viral protein containing complexes. With regard to changes in membrane architecture, viruses typically create membrane bound environments, called virus factories, to protect replication and assembly complexes from cellular defenses. At the electron microscopic level viroplasms are large virus factories that are amorphous structures containing virion particles, viral RNAs, and non-structural proteins, but typically exclude organelles. The term viroplasm was first used to describe such perinuclear virus factories produced by large DNA viruses and some (+) strand RNA viruses such as poxvirus and poliovirus. Recent research indicates that many plant infecting (+) strand RNA create microenvironments that are sometimes referred to as miniorganelles and these can range in size from vesicles or invaginations along organelle membranes to slightly larger virus factories. Typically these various membrane bound virus factories are induced by non-structural viral proteins and serve to concentrate replication proteins, viral genomes, and host proteins needed for efficient virus replication. Such extensive rearrangement of host membrane compartments are a hallmark of (+) strand RNA virus infection and the specific structures produces by various virus species have been reviewed in prior publications and will not be explored in depth here (Heath et al., 2001; Netherton et al., 2007; Wileman, 2007; Verchot, 2011).

The second cellular alteration mentioned above is the recruitment of host proteins, including cellular chaperones, to membrane bound sites required for virus replication and cell-to-cell movement. Among these are the heat shock protein (Hsp) 40, 70, 90, and 100 families of protein chaperones which are highly conserved across eukaryotes and are vital factors in the quality control of cellular proteins and protein complexes contributing to a wide range of cellular processes (Mayer, 2010). Chaperones within the context of the cellular quality control machinery enable misfolded or aggregated proteins to be refolded (Tyedmers et al., 2010) or targeted for degradation by cellular proteases (Bukau et al., 2006). The ubiquitin ligase machinery is central to ubiquitin tagging misfolded proteins and targeting them for degradation by cellular proteasomes. There are also reports that the ubiquitin ligase machinery is vital for regulating host immunity to infection. With regard to viral processes, there are few examples where the protein quality control machinery regulates viral proteins in the same way that it acts on cellular proteins. But there are also examples where viruses commandeer chaperones to become central components of replication complexes or drive virus egress into neighboring cells, providing activities that are outside of their normal cellular functions. Among these examples, it is not clear whether the entire machinery is dismantled or if there are an abundance of factors that can allow for some to be recruited without inhibiting normal cellular functions.

This review discusses the various contributions of cellular chaperones and folding enzymes, including a variety of Hsp, to the formation of viral multi-protein complexes. This article contrasts the cellular functions of such proteins to provide the reader with adequate information to consider whether cellular chaperones are acting within their normal context to enable viral protein folding, trafficking, and functioning, or whether they are diverted from their normal activities to provide novel contributions to virus infection. Understanding the various contributions of protein chaperones to cellular and viral activities could enable researchers to determine when and where such factors could be targeted by antiviral compounds to suppress disease. Given the rapid evolution of plant viral genomes and the slow evolution of Hsp proteins, it is reasonable to consider that therapeutic interventions targeting host components of the viral replication and transport machinery could offer a reliable approach to controlling disease.

## THE CONTRASTING ROLES OF CYTOSOLIC Hsp70 AND J-DOMAIN PROTEINS IN CELLULAR PROTEIN FOLDING AND PLANT RNA VIRUS INFECTION

**FIGURE 1:**
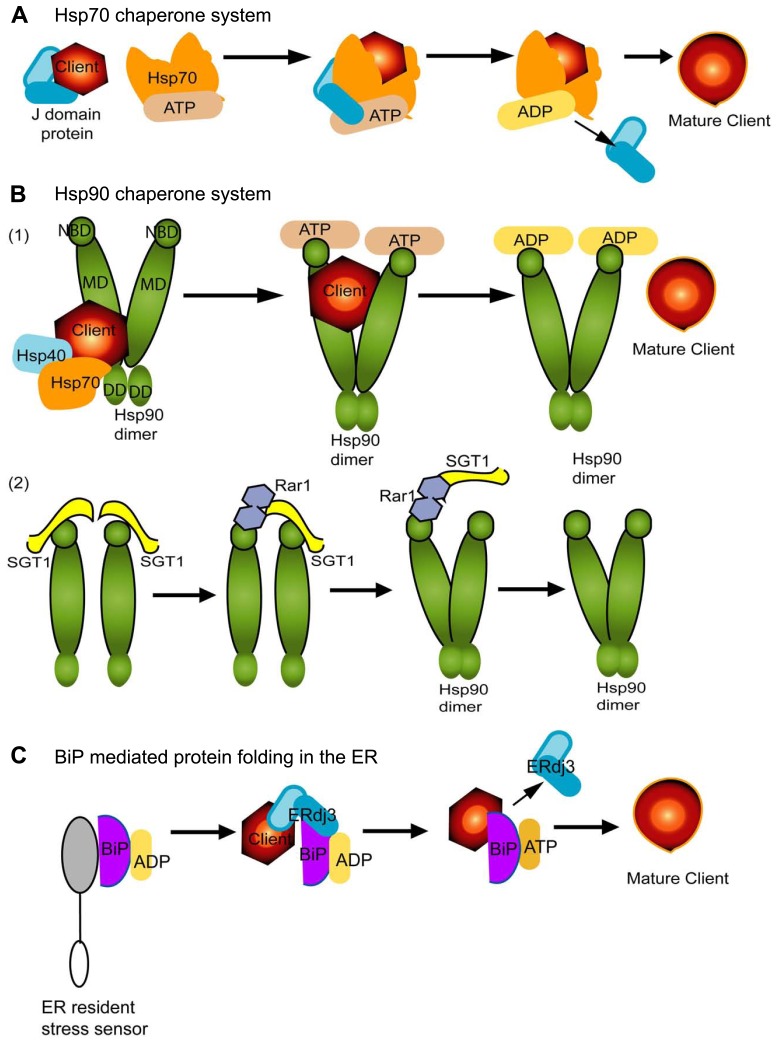
**The Hsp70, Hsp90, and BiP mediated protein folding systems are conserved across kingdoms and are vital contributors to plant virus infection and immunity.** Misfolded proteins can be referred to as “substrates” or “clients.” Hsp70 is shown in orange **(A,B)**. Hsp90 **(B)** is a dimer and has three domains which are represented in deep green and BiP **(C)** is shown in purple. Each chaperone in this figure depends upon ATP (beige) hydrolysis for client binding and release. ADP is depicted in yellow. J-domain proteins are a broad family of proteins that include Hsp40 and DNAJ-like homologs and are depicted in cyan in each panel. While each panel schematic shows a linear representation of the process for recruiting co-chaperones and clients for maturation, in fact the chaperone systems are dynamic and cycle between complex formation for maturation of a client followed, ATP hydrolysis, and disassembly. The cycles repeat in each example. **(A)** The J-domain protein binds to a misfolded protein client and delivers it to Hsp70. These proteins directly interact and it is ATP hydrolysis which enables the release of the J-domain protein. This is also followed by maturation and release of the client protein. **(B)** Hsp90 has a nucleotide-binding domain (NBD) at the N-terminus, the client and co-chaperone binding middle domain (MD), and the dimerization domain (DD) t the C-terminus. The NBD participates in ATP hydrolysis **(B1)** but, interestingly, also interacts with SGT1 and Rar1 **(B2)**. There are two types of co-factors represented in the figure: (1) Hsp40 and Hsp70 coordinate to recruit client proteins to Hsp90 dimers. The Hsp90 MD is primarily responsible for interactions with the misfolded client presented by the Hsp40/70 complex. ATP hydrolysis enables Hsp90 dimer conformational changes and client protein maturation. (2) Rar1-SGT1-Hsp90 are vital for folding and stabilization of NLR proteins. SGT1 and Rar1 are co-chaperones and function to assist the assembly of the Hsp90 dimer. The schematic shows the sequential binding and release of SGT1 and Rar1 to Hsp90. SGT1 binds to the ND domain of Hsp90. Two SGT1 proteins are drawn together bringing Hsp90 monomers into close proximity necessary for dimerization. Rar1 binds ND and interacts with SGT1, sequentially dissociating one and then the next SGT1. Thus, Rar1 enhances SGT1-Hsp90 interactions, but also aids dissociation of SGT1 from Hsp90. Thus the schematic attempts to represent the dynamics nature of their complex formation as proposed by Kadota et al. (2010). These associations are suggested by Shirasu (2009) to stabilize Hsp90 dimers for client substrate loading or release. **(C)** BiP is an Hsp70 homolog and vital contributor to the ERQC machinery. According to Kampinga and Craig (2010), an inactive BiP is bound to the ER luminal domain of a resident ER stress sensor, and to ADP. Upon recognition of misfolded proteins, ERdj3 is a J-domain protein with two domains for substrate and chaperone interactions. ERdj3 resides in the ER and recruits BiP and a misfolded client substrate into a complex. ADP conversion to ATP is necessary to release ERdj3 and subsequent client protein maturation by BiP in the ER.

Hsp70 family of proteins can interact with a wide range of cofactors and folding substrates and contribute to diverse biological processes. The most common cofactors are J-domain proteins (also known as Hsp40) which identify and recruit substrates to Hsp70 through direct interactions (**Figure 1A**). Nucleotide exchange factors (NEFs) are another set of cellular partners which stimulate dissociation of ADP and this fosters client dissociation upon refolding (**Figure 1A**; Kampinga and Craig, 2010). Thus, the Hsp70 chaperones cycle between substrate bound and free states and rely on the energy of ATP to induce conformation changes in the substrates (**Figure 1A**). Hsp70 can also partner with Hsp90 or Hsp100 (or ClpB) family to solubilize and refold protein aggregates into the native state (Mayer and Bukau, 2005; Kampinga and Craig, 2010; Tyedmers et al., 2010).

The Hsp70 and J-domain proteins are mentioned first because they are most often reported to associate with plant virus infection (Aparicio et al., 2005; Chen et al., 2008). Importantly, Hsp70 and J-domain proteins are not always linked in their contributions to plant virus infection which leads to the speculation that these factors can be diverted from their normal cellular functions to contribute to crucial viral protein complexes. Cytosolic Hsp70s play crucial roles in the replication cycle, intercellular transport, and virion assembly of many positive-strand RNA viruses including potexviruses, tobamoviruses, potyviruses, cucumoviruses, tombusvirus, and carmoviruses. Hsp70 gene expression is induced by these same positive-strand RNA viruses as well as by plant rhabdoviruses and tospoviruses, which have negative-strand genomes (Aparicio et al., 2005; Senthil et al., 2005; Chen et al., 2008; Wang et al., 2009; Mathioudakis et al., 2012). For some plant viruses, there is research knowledge concerning how viral proteins interact with Hsp70 and which aspects of virus infection are aided by these interactions, but for many viruses there is much to learn about the vital roles that Hsp70 plays in pathogenesis. For example, the inhibition of Hsp70 activity or expression alters the replication of turnip crinkle carmovirus although the exact role within the viral replication complex is not known (Chen et al., 2008). Beyond fulfilling key needs in viral pathogenesis, Hsp70 overexpression can enhance abiotic stress tolerance in plants. Thus, understanding the contrasting roles of Hsp70 in plant virus infection can be critical for designing broad strategies to control disease and improve plant tolerance to abiotic stresses. For example, diverting Hsp70 from its normal function could compromise abiotic stress responses. However, if viruses enhance Hsp70 gene expression or if there are multiple homologs or redundancy in function amongst homologs then it is possible that subversion of the Hsp70 machinery by the plant virus might have no impact on the normative cellular processes or might even serve to enhance abiotic stress tolerance. There is no data yet on this topic to know the impact of plant virus infection on Hsp70 related abiotic stress tolerance.

**FIGURE 2:**
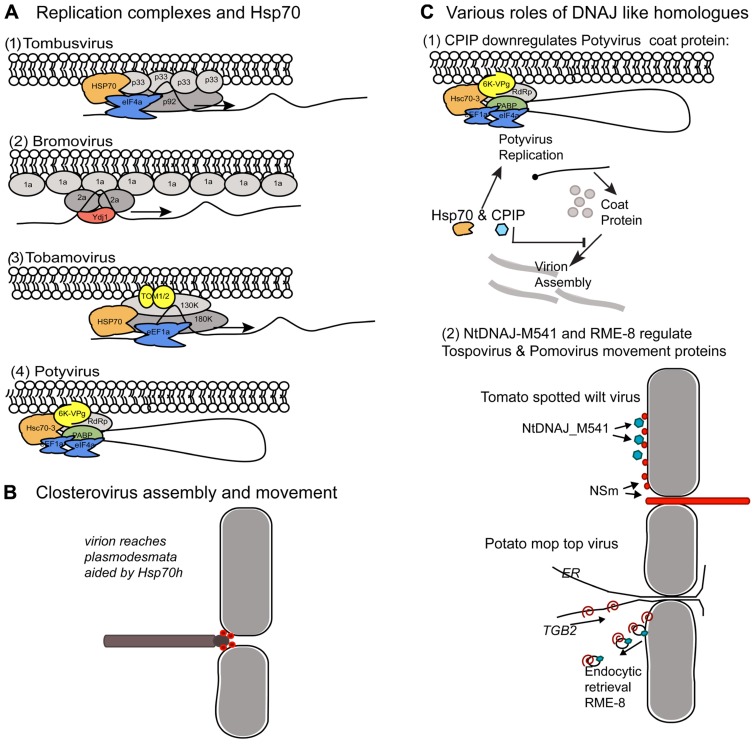
**Comparison of the host protein chaperones recruited to membrane bound viral complexes by unrelated (+) strand RNA viruses**. Similar chaperones provide different roles in the viral protein complexes. **(A)** Comparison of Hsp70 and Ydj1 interactions with various viral replicases. **(A1,2)** The tombusvirus and bromovirus replicases assemble in spherules along the peroxisome and ER membranes, respectively. **(A3,4)** Tobamoviruses and Potyviruses replicate in ER derived structures. **(A1)** Tombusviruses encode p33 and p92 proteins required for replication. The Hsp70 (orange) recruits p33 to cellular membranes. eIF4e (blue) is another cellular component of the replication complex. **(A2)** Bromoviruses encode 1a and 2a proteins. 1a forms a shell along the membrane. Ydj1p is a J-domain protein involved in negative-strand synthesis which interacts with the 2a protein. **(A3)** Tobamovirus replicase consists of the 130K and 180K proteins and accumulates on ER membranes. TOM1 and TOM2 are host proteins which provide membrane anchoring. Hsp70 and eEF1a are host factors that associate with the viral replicase. **(A4)** The potyvirus replicase is anchored to ER membranes by the viral encoded 6K-VPg. The host PABP, eEF1a, eIF4a, and Hsc70-3 proteins associate with the viral replicase. It is likely that the PABP brings the 3′ end of the genome near the 5′ end and that replication is initiated along a circular RNA. **(B)** Closterovirus virions are long filamentous particles with structurally differentiated tail domain. The viral movement protein is an Hsp70 homolog (Hsp70h; red spheres) which functions to both stabilize the tail region of the virion and aid trafficking across plasmodesmata. **(C)** Role of DNAJ homologs in regulating virus encapsidation and egress. **(C1)** Depiction of the Hsc70-3 containing viral replicase and its relationship to another Hsp70 and CPIP protein. This describes another role for Hsp70 in the potyvirus life cycle, unlike its role in replication depicted in **(A4)**. The Hsp70 and CPIP depicted here suppresses coat protein accumulation and blocks virion assembly. Virion assembly also serves to suppress viral genome translation and therefore, suppression of CP accumulation can enhance genome expression. This machinery reduces the impact of CP on viral genome translation. In this model, proposed by Hafren et al. (2010), CPIP recruits the potyvirus coat protein to Hsp70 which serves to aid ubiquitination and CP degradation. CPIP recruits the CP and thereby reduces its impact on viral genome translation. **(C2)** Yeast two-hybrid assays carried out using the tospovirus NSm protein identified NtDNAJ_M541 protein as an interacting partner. NSm (red spheres) associates with the plasma membrane, binds nucleocapsids, weakly binds RNA, and forms tubules. Given the myriad of NSm activities it is not yet clear how NtDNAJ_M541 (blue octagons) contributes to its functions. Pomovirus TGB2 movement protein is a transmembrane protein that resides in the ER and interacts with RME-8. TGB2 binds viral RNA and potentially cargoes it along the ER to plasmodesmata to facilitate intercellular transport. Researchers proposed that RME-8 is an endocytic marker indicating TGB2 is recycled from the plasma membrane back to the ER where it can bind viral genomes for further rounds of transport to the plasmodesmata.

Tombusvirus and bromovirus replication has been studied extensively using yeast as a host model system. Both viruses encode two protein components that comprise the core replicase. For Tomato bushy stunt virus and Cucumber necrosis virus (TBSV and CNV; tombusvirus) it is the p33 and p92 proteins. The p92 is produced by translational readthrough of the UAG stop codon at the end of the p33 domain. Both of the replicase proteins have membrane anchoring domains and assemble with viral RNA template along membrane sites (Panavas et al., 2005). Yeast Hsp70 and DNAJ homologs contribute to the assembly and activation of these viral replicases (**Figure 2A1**). Hsp70 plays a vital role in localizing the TBSV replicase to organellar membranes and in membrane insertion of the replication proteins *in vitro* and in *Nicotiana benthamiana* plants (Pogany et al., 2008; Wang et al., 2009). Two yeast Hsp70 proteins, named Ssa1p and Ssa2p, are present in purified TBSV replicase complexes and mutations in these genes cause cytosolic redistribution of the p33 and p92 replication proteins. With respect to Brome mosaic virus (BMV; bromovirus), the two viral protein components of the replicase are named 1a and 2a and are translated from separate genomic RNAs. The 1a multimerizes along endoplasmic reticulum (ER) membrane causing invaginations that lead to vesicle formation (Diaz et al., 2012). The 1a protein provides RNA capping and helicase activity. The 2a protein is the polymerase and recruits template RNA into the replication vesicles (Chen and Ahlquist, 2000). The yeast *Ydj1* encodes Ydj1p, which is a DNAJ homolog that normally interacts with the Ssa family of Hsp70, is vital for BMV replication (**Figure 2A2**) and interact with the 2a protein (Tomita et al., 2003). Mutations in *Ydj1* inhibit negative-strand RNA synthesis but do not inhibit 1a recruitment of 2a to membrane bound complexes. Thus, Ydj1p is proposed to play a role in the converting the complex to an active form that is capable of negative-strand RNA activation (Tomita et al., 2003). While Ydj1p, Ssa1p, and Ssa2p are separately identified as factors in these virus replication cycles, the co-chaperone complex itself has not be identified so it is not clear whether the individual factors are highjacked independently or if the entire chaperone complex is needed to drive membrane insertion and conformational changes in both systems.

Flock house virus (FHV) is primarily an insect infecting virus and is not defined as a plant virus, but is important to consider alongside TBSV and BMV because there are key similarities with regard to the viral replicases, and in laboratory experiments FHV replicates in plant cells as well as yeast and *Drosophila*. In yeast, FHV requires Ydj1p and Hsp70 chaperones for virus replication (Weeks and Miller, 2008). Single deletions of *Ssa1* or *Ssa2* did not alter FHV RNA3 accumulation in yeast but deletion of *Ssz1* which encodes an atypical Hsp70 resulted in an abundant increase in FHV RNA3 accumulation. However, deletion of the *Ydj1* gene suppressed FHV RNA replication while deletion of other DNA J homologs (*JJJ1, JJJ2*, or *ZUO1*) increased FHV RNA3 accumulation (Weeks and Miller, 2008; Weeks et al., 2010). The combined data show that the Ssa family of Hsp70 chaperones are essential for replication of several positive-strand RNA viruses in yeast. The fact that viruses which normally infect either plants or an insect commonly require of Hsp70 and Ydj1p is remarkable and suggests that the need for the Hsp70 complex for the replication of some positive-strand RNA viruses are maintained through evolution of their hosts. However, the mechanistic contribution to virus replication varies for each virus. For example, deletion of *Ssa1 *or *Ssa2* alters the membrane distribution of the tombusvirus replicase while similar deletions alter the post-translational stability of the FHV protein A polymerase (Weeks and Miller, 2008; Weeks et al., 2010). Thus, while the need for the Hsp70 complex for virus replication is well established, the mechanistic contributions cannot be broadly infer based on studies of a single plant virus.

Research conducted in plants has also identified Hsp70 associating with the tobamovirus and potyvirus replicase (**Figures 2A3,4**), although its role in these complexes is not yet described is such detail. Affinity purified Tomato virus mosaic virus (ToMV; tobamovirus) replicase identified Hsp70, eEF1A, TOM1, and TOM2A proteins associating with membrane bound complexes. TOM1 and TOM2A are integral membrane proteins normally associated with the vacuolar membrane but are highjacked by the ToMV replicase to the membrane site of virus replication (Nishikiori et al., 2006). Turnip mosaic virus (TuMV; potyvirus) RdRP co purifies with *Arabidopsis* Hsc70-3 and the poly(A) binding protein (PABP) in ER-derived vesicles (Dufresne et al., 2008).

Beyond aiding assembly of viral replication complexes, Hsp70 and DNAJ-like proteins contribute to virion assembly and cell-to-cell spread of viruses in other genera. Key examples of (+) strand RNA viruses include the potyviruses, closteroviruses, and pomoviruses. There is also evidence that the Hsp70 machinery contributes to the intercellular transport of (-) strand RNA genome containing tospoviruses.

With regard to viral coat protein (CP) interactions and virion assembly there are two examples. The first example is the closterovirus movement protein (MP) which is itself an Hsp70 homolog (Hsp70h). The Hsp70h plays dual roles in virion assembly and intercellular movement (**Figure 2B**) and its activities appear to be unlike the function of cellular Hsp70 that is depicted in **Figure 1**. Closteroviruses are filamentous viruses that have a long flexuous particles formed by the major capsid protein and a short tail formed by the minor capsid protein (CPm). For Citrus tristeza virus (CTV) and Beet yellows virus (BYV) Hsp70h combined with the viral encoded p61 protein enables the assembly of full-length virions by specifically enabling tail assembly by CPm (Satyanarayana et al., 2000, 2004; Alzhanova et al., 2007; Tatineni et al., 2010). The second function of Hsp70h is to traffic virions to plasmodesmata and enables intercellular transport. The closterovirus Hsp70h autonomously associates with the actin–myosin network and can move through plasmodesmata. Dominant negative mutants of class VIII myosins impede plasmodesmal localization of Hsp70h (Avisar et al., 2008). In *Cucurbita maxima*, Hsp70 homologs were identified to have the capacity to traffic through plasmodesmata and the combined data suggest that a subclass of Hsp70 chaperones engage the plasmodesmata trafficking machinery (Aoki et al., 2002). These data do not fit the current understanding of the roles for Hsp70 in protein folding and turnover, and suggests an alternative function in long distance trafficking that is worth further studying. It is reasonable to speculate the Hsp70 is a component of machinery that moves along the actin network or associates with myosins, but this topic requires further investigation to provide clear understand of this subclass of Hsp70 chaperones. However, these combined data of closterovirus Hsp70h and the *C. maxima* Hsp70s led researchers to speculate that there is a basic mechanism for filamentous virions to require chaperone activity to reach plasmodesmata and trigger viral RNA transfer to neighboring cells

Secondly, separate studies have reported the potyvirus CP interacting with Hsc70 and CPIP, which is a DNAJ-like protein (Hofius et al., 2007; Mathioudakis et al., 2012). In this example, the co-chaperone machinery appears to function in its normal role of client recruitment and modification. CPIP binds to the CP and delivers it to Hsp70 to aid ubiquitination and degradation (Hafren et al., 2010; **Figure 2C1**). Given the multimeric nature of CPs it is possible that the Hsp70 machinery ensures proper protein folding and prevents CP aggregation (Hafren et al., 2010). Beyond quality control regulation of the potyvirus CP, this mechanism also plays a significant role in regulating potyviral gene expression. Within the context of virus infection, the potyvirus CP functions to down-regulate viral gene expression and replication to enable genome encapsidation. As CPs build up in the cell, there becomes an increasing pressure toward suppressing viral genome expression and replication. Therefore, to prolong or increase the amount of genome translation and replication, the combined action of CPIP and Hsp70 serves to down-regulate CP-mediated effects on viral gene expression (**Figure 2C**; Hafren et al., 2010).

DNAJ proteins contribute to membrane bound events relating to virus intercellular movement and there are two well-studied examples (Soellick et al., 2000; **Figure 2C2**). First is RME-8, another DNAJ-like chaperone, which interacts with the pomovirus TGB2 MP (**Figure 2C2**). RME-8 localizes to endocytic vesicles and interacts with cytosolic Hsp70 to control clathrin-dependent endocytosis (Haupt et al., 2005). Thus, TGB2 may rely on endosome for recycling proteins to the cell’s interior where it can provide further rounds of transporting viral genomes from the site of replication to plasmodesmata. However, given the examples of TOM1 and TOM2A which are highjacked by TMV from the vacuolar membrane to viral replication complexes located on other membranes, it is possible that RME-8 is either a landmark for the endosome or plays a different role in TGB2 trafficking. Another example are the (-) strand RNA genome containing tospoviruses whose MP, named NSm, localizes to the plasma membrane and forms tubular extensions from the cell surface. Nsm also interacts with the nucleocapsid and genomic RNA and potentially functions to enable the tubule guided transport of the ribonucleoprotein complex across plasmodesmata. The tospovirus NSm MP interacts with a DNAJ-like protein (NtDnaJ_M541) from both *N. tabacum* and *A. thaliana* (Soellick et al., 2000; **Figure 2C2**). This factor belongs to a subclass of the DNAJ family that only contains the J-domain. Such factors contribute to protein translocation into the mammalian ER, plant peroxisomes, and microtubule formation. The particular role of the NtDnaJ_M541 protein is not known but researchers proposed that it either mediates Hsp70 dependent mechanism of virus movement or itself provides the motive force for ribonucleoprotein translocation to the plasmodesmata (Soellick et al., 2000).

## Hsp100 CHAPERONES REGULATE CELLULAR PROTEIN AGGREGATES BUT Hsp101 PROMOTES TOBAMOVIRUS TRANSLATION

The Hsp100/Clp family of chaperones belongs to the superfamily of AAA+ domain containing ATPases and some members act solely in the protein quality control network, functioning in protein disaggregation. This superfamily is defined by direct nucleotide binding and the presence of highly conserved Walker A and B motifs. Most AAA+ domain proteins form ATP bound oligomers and it is the molecular scaffold that is essential for HSp100/Clp as well as nucleotide-binding domain leucine-rich repeat (NLR) protein functions (Mayer, 2010; Bonardi et al., 2012). Hsp100 proteins can cooperate with the Hsp70-Hsp40 system to solubilize and refold aggregated substrate proteins (Zhang and Guy, 2005; Sharma et al., 2009). In plants, Hsp101 is required for thermotolerance and oxidative stress (Tonsor et al., 2008; Kim et al., 2012).

In TMV infection, Hsp101 and eIF4G are recruited by the 68 nucleotide 5′ untranslated leader, known as Ω, and enhances translation of the genomic RNA (Wells et al., 1998; Gallie, 2002). Other tobamoviruses such as Oilseed rape mosaic virus (ORSV) which lack the Ω sequence, do not display the Hsp101-dependent enhancement (Carr et al., 2006), which emphasizes the role of Ω in Hsp101 recruitment. Furthermore, the *N. tabacum* Hsp101 enhances translation of Ω-containing constructs in yeast. Genetic analysis of Hsp101 interactions with the TMV 5′ leader showed that it binds to a poly(CAA) sequence within Ω and aids the recruitment of eIF4F (Gallie, 2002). It is interesting that the Ω -Hsp101 enhancement is not conserved among all tobamoviruses. One possible explanation is that the Ω functions overlap with the 5′ cap and poly(A) tail for recruiting eIF4G (which is a subunit of eIF4F) to the mRNA. The Ω is more effective following heat shock and its presence can reduce the effectiveness of the 5′-cap and poly(A) tail for recruiting eIF4G (Wells et al., 1998). Given that the 5′ cap and poly(A) tail synergistically operate to recruit eIF4G, the Ω may not be crucial in all tobamoviruses and this could explain why it is not highly conserved across members of this genus (Carr et al., 2006).

## Hsp90 PLAYS ESSENTIAL ROLES IN HOST PLANT IMMUNITY AND VIRUS REPLICATION

Hsp90 is a highly conserved eukaryotic molecular chaperone. It contributes to the stabilization, or activation of proteins that are involved in signal transduction, protein trafficking, and immunity. Hsp90 typically forms a dimer and its associations with client proteins, as for Hsp70, are regulated by co-chaperones as well as ATP binding and hydrolysis (**Figure 1B1**). Its clients are often properly folded or are in a near native state. Hsp90 proteins have three functional domains: nucleotide-binding domain (NBD) at the N-terminus, the middle domain (MD) which is involved in client and co-chaperone binding, and the dimerization domain (DD) at the C-terminus (Zhang et al., 2010). Hsp90 forms an open homodimer mediated by interactions occurring through the DD domain. When the NBD binds ATP the N-terminal domains come into contact and for a closed conformation. Hsp90 cycles between open and closed conformations (**Figure 1B1**).

In mammalian cells, Hsp40, Hsp70, and Hsp90 are known to cooperate in the maturation of certain client proteins (**Figure 1B1**). The Hsp90 machinery also associates with ubiquitin-dependent degradation processes that contribute to immune regulation (Zhang et al., 2010). In plants and animals, *Hsp90*, *SGT1* (suppressor of G2 allele of skp1) and *Rar1* are essential to the function of many NLR proteins (**Figure 1B2**). NLR proteins are pathogen sensors which contribute to host immunity by activating disease defense responses (Azevedo et al., 2002; Shirasu, 2009; Kadota et al., 2010; Kadota and Shirasu, 2012). Mutations in *Hsp90* can lead to the loss of NLR-mediated defense responses in plants. Most NLR proteins exist in a cell in a near native state but upon recognition of a pathogen effector the NLR proteins are folded and may form dimer or multimeric complexes that are necessary for immune regulation. The Hsp90–SGT1–Rar1 machinery is needed for *Rx*- or *N*-mediated resistance to Potato virus X (PVX) or TMV (Boter et al., 2007; Takabatake et al., 2007), *RPM1* or *RPS2* resistance to *Pseudomonas syringae* (Cai et al., 2006; Kadota et al., 2010), *Mi-1* resistance to root knot nematodes (Bhattarai et al., 2007), *Mla*-resistance to powdery mildew in barley (Hein et al., 2005) among others. The Hsp90–SGT1–Rar1machinery is intriguing because the required partnership among these three factors differs from the Hsp40–Hsp70–Hsp90 partnership for client recruitment and folding. **Figure 1B2** shows that SGT1 and Rar1 bind to the N-terminal ATPase domain of Hsp90, but do not promote ATP hydrolysis. Instead, Rar1 enhances SGT1–Hsp90 interactions and form an asymmetric complex that holds the Hsp90 dimer to enable loading or release of the client protein. Both *Rar1* and *SGT1* are required for steady state accumulation of many NLR proteins and SGT1 plays an addition role in recruiting the NLR client to Hsp90 (Kadota et al., 2008, 2010; Shirasu, 2009; Kadota and Shirasu, 2012). This model is substantiated by yeast two-hybrid assays showing SGT1 and Rar1 proteins interact in the absence of Hsp90. Also mutations in Rar1 can reduce NLR protein accumulation but the consequence is not as significant as mutations affecting SGT1 and Hsp90. Thus, the NLR stability is mediated by SGT1–Hsp90 complex and enhanced by the presence of *Rar1*.

The SGT1–Hsp90–Rar1 machinery is particularly intriguing because each of these factors provide additional roles in biological processes that are independent of each other (Shirasu and Schulze-Lefert, 2003). *Rar1* was shown in soybean and *Arabidopsis* to be essential for the induction of pathogenesis-related (PR) gene expression and contributes to PAMP-mediated immunity (Fu et al., 2009). While *SGT1* is required for *Rx*-steady state accumulation, it also negatively regulates some NLR proteins in *Arabidopsis* and helps to mediate systemic acquired resistance in soybean (Boter et al., 2007; Fu et al., 2009). With regard to plant virus infection, *SGT1* is specifically induced by SMV, PVX, and Plantago asiatica mosaic virus (PlAMV) infection in susceptible hosts (Komatsu et al., 2010; Ye et al., 2012b). PlAMV and PVX are both potexviruses and VIGS silencing *SGT1* enhanced virus accumulation relative to leaves treated with only the VIGS vector. *N. benthamiana* plants overexpressing SGT1 show enhanced systemic accumulation of PVX (Ye et al., 2012b). Unlike PVX, PlAMV infection causes systemic necrosis and silencing *SGT1* and *RAR1* reduces these symptoms (Komatsu et al., 2010). Thus, for potexviruses it appears that SGT1 contributes to the regulation of systemic virus accumulation. Moreover, SGT1 associates with *Hsc70 *in Arabidopsis and this interaction contributes to basal resistance. Given that *Hsc70* is also a positive factor in plant virus infection this story may be quite complicated. More research is needed to uncover the various roles of SGT1 as a co-chaperone in events that modulate immunity and promote plant virus multiplication.

**FIGURE 3:**
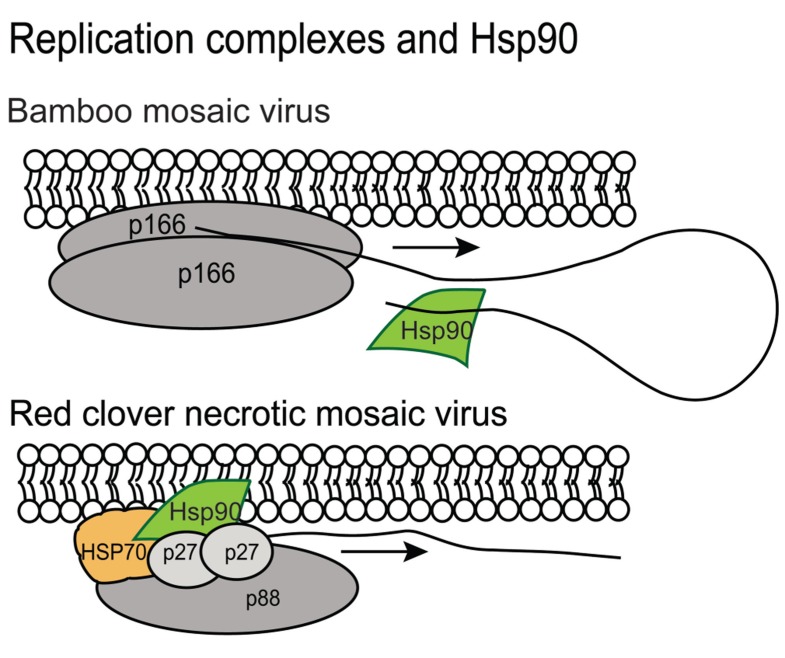
**Hsp90 contributes to the BaMV and RCNMV replication complexes**. The gray spheres represent the membrane bound viral components of the replicase. The BaMV p166 protein is represented as a dimer. The RCNMV p88 and p27 are also represented. Hsp90 is indicated in green and binds to the 3′ end of the BaMV genome. It is not known to interact with other viral genomes making this observation unique. Hsp90 and hsp70 are also components of the RCNMV replicase and are essential for membrane recruitment of the complex.

There are also reports showing Hsp90 plays a positive role in Bamboo mosaic virus (BaMV; a potexvirus), Red clover necrotic mosaic virus (RCNMV; a dianthovirus), and FHV infection and it appears to provide different partnerships with the various viral replicases (**Figure 3**). The *N. benthamiana* Hsp90 was reported by Huang et al. (2012) to interact specifically with the 3′ untranslated region of BaMV. Hsp90 does not associate with the 3′ region of the BaMV-associated satellite RNA, PVX (potexvirus genus) or Cucumber mosaic virus (CMV; cucumovirus genus) genomic RNAs suggesting that this is a unique interaction that promotes BaMV replication (**Figure 3**; Huang et al., 2012). In contrast, RCNMV encodes two replication associated proteins named p27 and p88. Hsp70 and Hsp90 interact with p27 and lead to the recruitment of RNA 2 to the ER. Hsp70 and Hsp90 also promote translation of p27 (Mine et al., 2010, 2012). FHV also requires Hsp90 for assembly of the viral replication complex and protein-A accumulation (Kampmueller and Miller, 2005). These very recent discoveries that Hsp90 contributes to the initiation of viral RNA synthesis in a virus species-specific manner is intriguing. While the interactions between component proteins of the viral replicase with Hsp90 seem to have little in common, it is possible that a common fold in the proteins is recognized by Hsp90 or that a viral RNA element first attracts Hsp90 which then recruits other of viral replication factors (Huang et al., 2012). Further investigations are needed to unlock the mechanism of replicase assembly and the role of Hsp90 for these and other plant viruses.

## ER CHAPERONE SYSTEM AND ITS EMERGING IMPORTANCE IN PLANT VIRUS INFECTION

A separate set of chaperones and folding enzymes exist in the ER and contribute to the ER quality control (ERQC) machinery which regulates the folding of newly synthesized proteins (Meunier et al., 2002). Proper folding and assembly is necessary for proteins entering the secretory pathway to reach their appropriate cellular destinations (Iwata and Koizumi, 2012). Key components of the ERQC include protein disulfide isomerase (PDI) which enables the formation of disulfide bonds in proteins, calreticulin (CRT) and calnexin (CNX) which are lectin-like chaperones that recognize and monitor N-linked glycan modifications (Meunier et al., 2002; Meusser et al., 2005), and the ER luminal-binding protein BiP, which is a member of the Hsp70 family that monitors protein folding and maturation in the ER. Glycoproteins can also be processed by ERp57 (a member of PDI family) which enable the formation of disulfide bonds (Ellgaard and Helenius, 2001, 2003).

Both the CRT/CNX and BiP chaperone systems sequester malformed proteins in the ER for refolding. N-linked glycosylation occurs through the transfer of a triglucosylated, branched chore oligosaccharide to a nascent polypeptide. The core oligosaccharide is trimmed by ER resident glucosidases to the monoglucosylated form. CNX and CRT recognize N-linked glycans attached to proteins which function to ensure the glucose is removed from the glycan. Improperly trimmed glycans can go through a reiterative process of transfers between the glucosidases and CNX/CRT to ensure proper maturation prior to ER export. BiP resembles other Hsp70 proteins in that its interactions with cofactors and substrates are regulated by the ATPase cycle (**Figure 1**). In this system ERdj3, which is a member of the Hsp40 family, first binds to the unfolded proteins and recruits BiP (Kampinga and Craig, 2010). The binding and release of nascent chains is controlled by the cycle of ATP and ADP exchange (**Figure 1**). Similar to CRT/CNX system, BiP undergoes cylces of binding and release from unfolded proteins. Co-chaperones include ERdj3 which is an Hsp40 and PDI. When proteins fail to mature properly, they are directly cleared from the ER and degraded by the ubiquitin-proteasome system.

CRT also functions in Ca^2^^+^ sequestration and in plants, accumulates in plasmodesmata. CRT interacts directly with the TMV MP and is suggested to play a regulatory role in promoting virus intercellular transport. Overexpression of CRT interferes with TMV cell-to-cell movement and is suggested to direct TMV MP from plasmodesmata to microtubules. It is worth speculating that this could lead to TMV MP degradation (Chen et al., 2005). Interestingly, BiP, CRT, and PDIs including ERp57 are up-regulated during *N*-mediated resistance to TMV (Caplan et al., 2009). Silencing ERp57, CRT2, and CRT3 in *N*-gene expressing *N. benthamiana* led to partial restoration of systemic accumulation lending further support to earlier reports that up-regulating CRT blocks TMV movement. CRTs also regulate the folding of plasma membrane localized induced receptor-like kinase (IRK) that functions during *N*-mediated resistance (Caplan et al., 2009).

**FIGURE 4:**
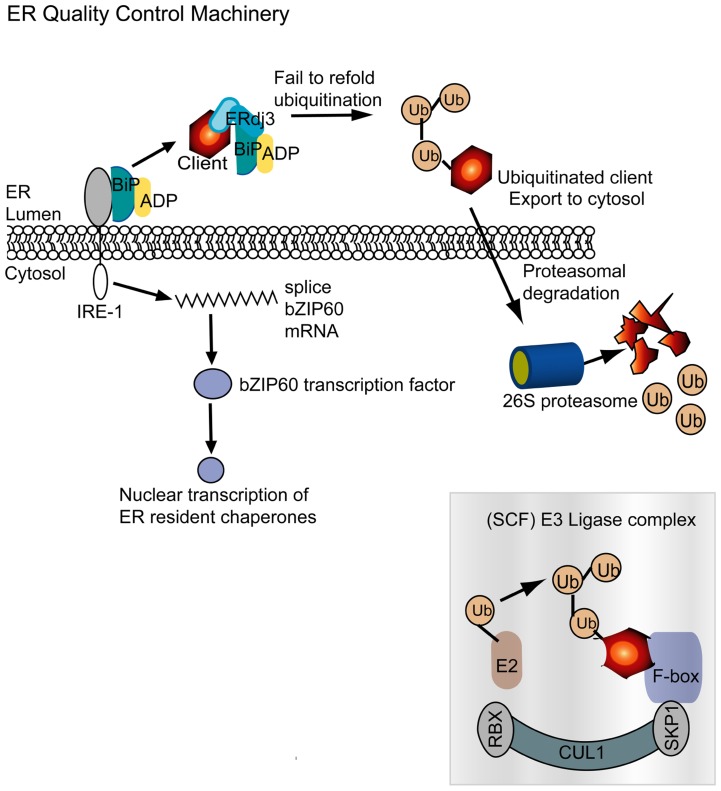
**The ER quality control machinery monitors protein folding in the ER following translation**. BiP is an ER chaperone that in its resting state is bound to the ER stress transmembrane receptor IRE-1 and to ADP. A nascent or misfolded protein is identified and recruited by ERdj3, which is an Hsp40 homolog, and BiP is redirected to this complex. As seen in **Figure 1**, the role of BiP is to refold misfolded proteins. But in the event that this does not succeed, the misfolded client is ubiquitinated in the ER. Hrd1p/Der3p in mammals, yeast, and plants is responsible for substrate ubiquitination in the ER (Meusser et al., 2005; Zhang and Kaufman, 2006; Su et al., 2011). The ubiquitinated substrates degraded in the cytosol by the 26S proteasome. The SCF E3 ubiquitin ligase complex, depicted here, is a cytosolic complex often described to be associated with the plant NLR immune system. It is intriguing to see that plant viruses require or mimic components of the SCF complex. IRE-1 senses accumulation of misfolded proteins and splices bZIP60 mRNA. bZIP60 is a transcription factor that upon translation is transported to the nucleus and activates expression of ER resident chaperones, including BiP. Up-regulation of the network is designed to restore ER homeostasis by eliminating malformed proteins.

BiP is best known for its central role in ER stress and the unfolded protein response (UPR; **Figure 4**). In the absence of ER stress, BiP binds to the ER luminal domain of IRE1. Upon stress, BiP moves away from IRE1 and binds to the unfolded protein. IRE1 possesses a cytosolic endoribonuclease domain which is activated by the uncoupling of BiP and IRE1 (Iwata and Koizumi, 2012; Parmar and Schroder, 2012). IRE1then cleaves exon–intron junctions in the mRNA encoding the bZIP60 transcription factor. The bZIP60 is translocated to the nucleus where it activates expression of genes involved in the UPR. Overexpression of BiP suppresses the UPR because it increases the amount of protein binding IRE1 and enabling protein maturation. The PVX TGB3 protein is essential for virus movement and is an ER resident protein that appears to stimulate expression of the IRE1-major downstream effector *bZIP60 *as well as BiP and CRT (Ye et al., 2011). Silencing bZIP60 also impairs PVX accumulation in protoplasts, indicating that activation of UPR related transcription factor is vital for PVX infection. Overexpression of TGB3 from a CaMV 35S promoter or from a TMV vector can cause HR-like lesions *N. benthamiana* leaves. Experiments also revealed that BiP plays a cytoprotective role in virus infected leaves and its overexpression can alleviate TGB3 or virus-induced cell death. These data argue that BiP and the UPR components of a pro-survival response that is activated by TGB3 to create a cellular environment that enables the spread of virus infection.

Another component of the ERQC machinery that works in conjunction with the ER resident chaperones is a mechanism to eliminate the malformed proteins from the ER. Such malformed proteins that cannot be refolded are recognized by Hrd1/Der3p which acts to ubiquitinate substrates to enable their dislocation and subsequent degradation. Hrd1p/Der3p resides in the ER and provides the essential ubiquitin ligase activity that precedes proteolytic breakdown of misfolded proteins. Hrd1p/Der3p is well described in mammal and yeast cells and was recently identified in plant cells (Meusser et al., 2005; Zhang and Kaufman, 2006; Su et al., 2011). The ubiquitinated substrates are transported out of the ER for degradation by the 26S proteasome.

Curiously there are other ubiquitin ligases that interact with the 26S proteinase, including SCF E3 ubiquitin ligase complex (**Figure 4**). This complex includes the co-chaperones SKP1 and Cullin. While TGBp3 is not known to induce Hrd1p/Der3p it has been shown to activate expression of SKP1 (Ye et al., 2011). Other plant viruses that are known to directly interact with SKP1 include the polerovirus P0 silencing suppressor protein and the nanovirus Clink. Both P0 and Clink have F-box like domains that can interact with SKP1 (Aronson et al., 2000; Pazhouhandeh et al., 2006; Bortolamiol et al., 2007). Up-regulation of SKP1 by PVX TGB3 or SKP1 interaction with P0 or Clink lead to enhanced virus accumulation. These combined data suggest that certain plant viruses stimulate chaperones or co-chaperones involved in protein turnover as a means to create a favorable environment for efficient replication. These factors might rely on SKP1-dependent machinery to degrade host factors that could impede replication or movement and may be involved in immunity.

## CONCLUSION

This article provides examples where plant viruses subvert a few key cellular chaperones and cofactors from their normal cellular function into viral protein complexes and examples where certain chaperones are likely to function within their normal cellular context, and viral proteins are the recognized substrates. With regard to subversion of chaperones, the tombusvirus, tobamovirus, and potyviruses require Hsp70, while BaMV and dianthoviruses require Hsp90 to participate early in the formation of active membrane anchored replication complexes (Nishikiori et al., 2006; Wang et al., 2009; Hafren et al., 2010; Huang et al., 2012; Mine et al., 2012). It is possible that these viral replicases have a common fold that can be recognized by the Hsp70 or Hsp90 partner, however future research is needed to better understand how these two cellular chaperones participate in the replication of a wide range of unrelated viruses. *Ydj1, JJJ*, *JJ2*, and *ZOU1 *are *Hsp40* homologs in yeast that act independently of Ssa1/2 to enable replication of either BMV or FHV (Tomita et al., 2003; Weeks and Miller, 2008; Weeks et al., 2010). The combined data among different plant viruses indicate that the mechanistic contributions of chaperones to RNA virus replication varies among viruses and that there is not one broad definition of how these viral replicases assemble with host factors. The requirement for *Ydj1* appears to be uncoupled from *Hsp70* for BMV replication. However, we do not yet understand whether these Hsp70 and Hsp40 separately or in combination regulate the folding and assembly or membrane anchoring of the viral replicases. It worth speculating that Hsp chaperones aid in recruiting essential host factors such as the PABP, eEF1a, or eIF4a into the replication complex or to stabilize associations between viral proteins that comprise the replication complexes (Leonard et al., 2004). They may also provide stability to the membrane anchor for the replication complex. Evidence that Hsp90 interacts with 3′ end of the BaMV RNA indicates that chaperones may be subverted to stabilize viral RNA. Furthermore, there is no evidence yet to indicate whether the ATPase activities are essential for the chaperone functions within the viral replication complexes (Huang et al., 2012). In summary, further research is needed to understand mechanistic contribution of these factors to virus replication. With regard to the roles of *Hsp70* and virus movement, there is evidence that Hsp70 overexpression enhances virus movement but mechanistically we know very little about its role in the plasmodesmata or interactions with viral MPs. It is intriguing that the closterovirus MP is an Hsp70 homolog which functions to stabilize the virion as well as direct plasmodesmata trafficking. Perhaps further studies with this protein will provide insight into the activities Hsp70 contributes to intercellular trafficking.

Ydp1, CPIP, RME-8, and NtDNAJ_M541 are J-domain proteins that are subverted by the plant viruses. Except for Ydp1 which associates with the BMV replicase, most interact with structural or MPs. CPIP is described as a factor that down-regulates the potyvirus CP, RME-8 is involved in endocytic trafficking of the pomovirus TGB2 protein from the plasma membrane toward the cell interior, and the role of the NtDNAJ_M541 protein is not yet defined (Haupt et al., 2005; Hafren et al., 2010). These factors are intriguing because they are known to identify client proteins for partner chaperones. Thus, it is easy to imagine that they might identify the viral proteins clients, relying on their substrate binding sites for stabilizing interactions. The questions we are left with, is whether these proteins are highjacked by the virus and subverted for viral functions, or if they target these viral proteins for degradation. For example, we do not yet know the next step in CPIP led processes, but researchers proposed that CPIP targets the CP for degradation (Hafren et al., 2010). This activity promotes viral genome expression and replication but down regulates encapsidation. The tospovirus NSm protein is required for virus intercellular movement but the mechanism for virus transport is not fully understood. Nsm accumulates along the plasma membrane, forms tubules, and interacts with a viral ribonucleoprotein complex which is transported between cells. NtDNAJ_M541 might stabilize the tospovirus Nsm in tubules or in the plasma membrane, and it might also stabilize complexes involving the capsid and genomic RNA (Soellick et al., 2000). On the other hand, this DNAJ-like protein could target Nsm for degradation to alleviate stress on the plasma membrane or modulate the size of the tubules. The function of RME-8 interactions with the pomovirus TGB2 protein is also uncertain. Pomoviruses encode three MPs known as the “triple gene block” proteins. TGB2 binds viral RNA, inserts into the ER network and might be responsible for trafficking viral RNA toward the plasmodesmata. Upon delivering the genome cargo to its destination TGB2 might move along the plasma membrane where it is recycled back to the cells interior by the endocytic machinery for further rounds of RNA trafficking. It is also possible that TGB2 is directed by the endosome to the vacuole for degradation. Thus RME-8 might function as a chaperone either to stabilize and regenerate movement complexes, or aid in protein turnover. Thus, considering that we know so little about CPIP, NtDNAJ_M541, and RME-8 in viral processes, future research is likely to produce fascinating new insights into the machinery regulating viral encapsidation and egress.

Finally, the potexvirus TGB3 protein up-regulates the expression of ER resident chaperones via bZIP60 transcription factor indicates that the ERQC machinery plays a vital role in plant virus infection (Ye et al., 2011, 2012a). This is the first example of a plant viral MP that activates bZIP60 to induce host gene expression. Up-regulation of ERQC machinery could function to stimulate protein folding and maintain ER homeostasis during plant virus infection. This might be a necessary activity to promote virus replication and spread. It is also possible that TGB3 identifies host proteins, such as NLR proteins for degradation via the proteasome. The SCF E3 ligase complex contains SKP1 and F-box protein to aid client protein ubiquitination prior to proteasomal degradation. Given a report by Ye et al. (2011) that SKP1 is induced by TGB3 and is important for virus infection it is possible that the PVX TGB3 protein acts at the ER to redirect such factors to the E3 ligase complex for ubiquitin-proteasome pathway soon after translation as a means to block host immune responses (Ye et al., 2012a). Further research is needed to identify factors that are degraded in a manner that is TGB3 dependent.

While the above discussion of Hsp40, Hsp70, and Hsp90 supports the hypothesis that the chaperone machinery is somewhat dismantled and reconfigured to support virus replication, the newer data concerning CPIP, RME-8, and NtDNAJ_M541, and the ERQC could be viewed as evidence that the chaperone machinery is diverted to recognizing alternative substrates while their cellular functions are unaltered. In other words, the endocytic machinery and ERQC machinery appears to remain intact but the virus piggybacks onto the machinery to achieve a successful infection. This is important to consider because it could represent a manner in which the virus can evade defense within the host and avoid recognition by the immune system. Alternatively, we know so little about how plant viruses interact with the endocytic machinery or ERQC machinery but we do know that both systems can achieve protein degradation. Endosomes containing viral components could fuse with the vacuole to degrade viral components and ERQC machinery to down-regulate infection by identifying viral proteins as foreign or aberrant products that need to be degraded through the proteasome. Thus, these machineries could be part of an immune response. We do know from animal virus research that RNA viruses have mechanisms to cleverly evade recognition by the host immune system and this can include interacting with the host autophagic machinery in a manner that promotes infection. Future research is likely to examine the exciting possibilities that the ERQC machinery or endocytic machinery are natural extensions of the antiviral defense machinery or essential pathway to achieving successful infection. Understanding their roles in infection could be quite valuable for designing strategies for controlling virus disease which target host machinery that is vital for infection.

## Conflict of Interest Statement

The author declares that the research was conducted in the absence of any commercial or financial relationships that could be construed as a potential conflict of interest.
